# Effectiveness of nursing case management versus usual care for blood pressure control in adults with hypertension: a systematic review

**DOI:** 10.17533/udea.iee.v39n1e04

**Published:** 2021-03-05

**Authors:** Maria de Fátima Mantovanil, Luciana Puchalski Kalinke, Ângela Taís Mattei da Silva, Juliana Perez Arthur, Cremilde Aparecida Trindade Radovanovic, Carina Bortolato-Major

**Affiliations:** 1 . Nurse, Ph.D. Universidade Federal do Paraná, Curitiba, Paraná- Brazil. Email: mariadefatimamantovani@gmail.com Universidade Federal do Paraná Universidade Federal do Paraná CuritibaParaná Brazil mariadefatimamantovani@gmail.com; 2 . Nurse, Ph.D. Universidade Federal do Paraná, Curitiba, Paraná - Brazil. Email: lucianakalinke@yahoo.com.br Universidade Federal do Paraná Universidade Federal do Paraná CuritibaParaná Brazil lucianakalinke@yahoo.com.br; 3 . Nurse, Ph.D. Universidade Federal do Paraná, Curitiba, Paraná- Brazil. Email: angelataismattei@gmail.com Universidade Federal do Paraná Universidade Federal do Paraná CuritibaParaná Brazil angelataismattei@gmail.com; 4 . Nurse, M.Sc. Universidade Federal do Paraná, Curitiba, Paraná- Brazil. Email: julianaperez4@gmail.com.br Universidade Federal do Paraná Universidade Federal do Paraná CuritibaParaná Brazil julianaperez4@gmail.com.br; 5 . Nurse, Ph.D. Universidade Estadual de Maringá, Maringá, Paraná-Brazil. Email: kikanovic2010@hotmail.com Universidade Estadual de Maringá Universidade Estadual de Maringá MaringáParaná Brazil kikanovic2010@hotmail.com; 6 . Nurse, Ph.D. Universidade Estadual do Norte do Paraná, Paraná-Brazil.E-mail: cabortolato@uenp.edu.br Universidade Estadual do Norte do Paraná Universidade Estadual do Norte do Paraná Paraná Brazil cabortolato@uenp.edu.br

**Keywords:** adult, case management, hypertension, nursing care, patient care planning, primary health care, systematic review, adulto, manejo de caso, hipertensión, atención de enfermería, planificación de atención al paciente, atención primaria de salud, revisión sistemática, adulto, administração de caso, hipertensão, cuidados de enfermagem, planejamento de assistência ao paciente, atenção primária à saúde, revisão sistemática

## Abstract

**Objective.:**

To synthesize the best available evidence regarding the effectiveness of nursing case management in primary health care, compared to usual care, in improving blood pressure in adults over 18 years with hypertension.

**Methods.:**

Systematic review that includes studies carried out with adult patients diagnosed with hypertension, with or without other concomitant chronic diseases, followed-up by a case manager nurse, who evaluated the effectiveness of case management in the improvement of blood pressure. A critical evaluation of the studies was made and the results of interest were described using the instruments and tools from the Joanna Briggs Institute. Due to the heterogeneity of the included studies, the results of similar measures were not grouped in statistical meta-analysis. A narrative and tabular format was used to synthesize and present them.

**Results.:**

Six randomized controlled trials were critically evaluated and included in the review. The total sample was 1963 participants. The results showed the outcomes compared at baseline and at the end of follow-up (six or twelve months). Regarding the main outcome, systolic and diastolic blood pressure, there was some reduction in the group followed-up through case management in studies lasting six months; however, the impossibility of comparing the findings poses limitations to answering the questions in this review.

**Conclusions.:**

Despite the heterogeneity of the studies, the narrative and tabular analysis demonstrated that short-term case management in primary care (six-month studies) helped to reduce blood pressure levels, although the level of evidence for these results is low or very low.

## Resumen

**Objetivo.:**

Sintetizar las mejores evidencias disponibles sobre la efectividad del manejo de casos de enfermería en la atención primaria en salud en comparación con los cuidados habituales para mejorar la presión arterial en adultos mayores de 18 años con hipertensión arterial sistémica.

**Métodos.:**

Revisión sistemática de estudios realizados en pacientes adultos con diagnóstico de hipertensión arterial, monitoreados por el enfermero gestor de casos quien evaluó la efectividad del manejo de la mejoría de la presión arterial con o sin otras enfermedades crónicas concomitantes. Se realizó una evaluación crítica de los estudios y se describieron los resultados de interés utilizando los instrumentos y herramientas del Instituto Joanna Briggs. Debido a la heterogeneidad de los estudios, los resultados de medidas similares no se agruparon en el meta-análisis estadístico. Se utilizó un formato narrativo y tabular para sintetizarlos y presentarlos.

**Resultados.:**

Seis ensayos clínicos aleatorios fueron evaluados críticamente e incluidos en la revisión. La muestra total fue de 1963 participantes. Los resultados mostraron los hallazgos comparados al inicio y al final del seguimiento (seis o doce meses). En cuanto al resultado principal, la presión arterial sistólica y diastólica tuvo una reducción en el grupo acompañado por el gestor de manejo de casos en los estudios que duraron seis meses; sin embargo, la imposibilidad de comparar los hallazgos plantea limitaciones para responder las preguntas de esta revisión.

**Conclusión.:**

A pesar de la heterogeneidad de los estudios, el análisis narrativo y de las tablas demostró que la gestión de casos por enfermería, a corto plazo (estudios de seis meses), en atención primaria, ayudó a reducir los niveles de presión arterial, aunque el nivel de evidencia para estos resultados fue bajo o muy bajo.

## Resumo

**Objetivo.:**

Sintetizar as melhores evidências disponíveis sobre a efetividade do gerenciamento de casos de enfermagem na atenção primária em saúde, em comparação com os cuidados usuais na melhoria da pressão arterial em adultos acima de 18 anos com hipertensão arterial sistêmica.

**Métodos.:**

Revisão sistemática que incluiu estudos realizados com pacientes adultos diagnosticados com hipertensão, com ou sem outras doenças crônicas concomitantes, acompanhados por enfermeiro gestor de casos, que avaliou a eficácia do gerenciamento de casos na melhora da pressão arterial. Foi realizada uma avaliação crítica dos estudos e descritos os resultados de interesse com a utilização dos instrumentos e ferramentas do Instituto Joanna Briggs. Devido à heterogeneidade dos estudos incluídos, os resultados de medidas semelhantes não foram agrupados na meta-análise estatística. Um formato narrativo e tabular foi usado para sintetizá-los e apresentá-los.

**Resultados.:**

Seis ensaios clínicos randomizados foram avaliados criticamente e incluídos na revisão. A amostra total foi de 1963 participantes. Os resultados mostraram os desfechos comparados no início e no final do acompanhamento (seis ou doze meses). Em relação ao desfecho principal, pressão arterial sistólica e diastólica, houve alguma redução no grupo acompanhado pelo gerenciamento de casos em estudos com duração de seis meses; no entanto, a impossibilidade de comparar os achados impõe limitações para responder às perguntas desta revisão.

**Conclusões.:**

Apesar da heterogeneidade dos estudos, a análise narrativa e tabular demonstrou que o gerenciamento de casos de curto prazo na atenção primária (estudos de seis meses) ajudou a reduzir os níveis de pressão arterial, embora o nível de evidência para esses resultados seja baixo ou muito baixo.

## Summary of Findings

**Table:** 

Case management compared to usual care for hypertensive adults in primary care	Case management compared to usual care for hypertensive adults in primary care	Case management compared to usual care for hypertensive adults in primary care
Bibliography: Effectiveness of nursing case management versus usual care for blood pressure control in adults with hypertension: a systematic review	Bibliography: Effectiveness of nursing case management versus usual care for blood pressure control in adults with hypertension: a systematic review	Bibliography: Effectiveness of nursing case management versus usual care for blood pressure control in adults with hypertension: a systematic review
Systolic blood pressure (6 months)	695 (4 RCTs)	⨁◯◯◯ VERY LOW^a,b,c^
Diastolic blood pressure (6 months)	695 (4 RCTs)	⨁⨁◯◯ LOW^a,c^
Systolic blood pressure (12 months)	1216 (2 RCTs)	⨁⨁◯◯ LOW^e,d^
Diastolic blood pressure (12 months)	1216 (2 RCTs)	⨁⨁⨁◯ MODERATE^e^

Explanations: (a) Risk of bias: downgraded once because one article scored 11/13, one scored 9/13, and two scored below 9 (JBI-SUMARI appraisal score); (b) Inconsistency: downgraded twice because heterogeneity was over 75%; (c) Inaccuracy: downgraded once due to sample size; (d) Inconsistency: downgraded once due to heterogeneity between 50 and 75%; and (e) Risk of bias: downgraded once because one article scored 11/13 and another article scored 7/13 (JBI-SUMARI appraisal score).

## Introduction

Hypertension is one of the main risk factors for cardiovascular diseases (CVD). In recent years, progress has been made in its treatment, but the number of hospitalizations with important socioeconomic costs remains high.(1) Worldwide, hypertension affects 22% of people over 18 years of age.(2) In Brazil, data from the Surveillance of Risk and Protection Factors for Chronic Diseases by Telephone Survey (Vigitel) in 2018 pointed out that the prevalence of adults who reported having a medical diagnosis of hypertension was 24.7%, with frequency increasing with age in both sexes and in the less educated part of the population.(3)

Hypertension represents a public health problem due to its numerical magnitude and potential to cause damage to the population. In view of this context, research about hypertension aimed at enhancing prevention and control actions arising from the health policies of local governments is sorely needed. This health condition is considered a silent threat because it does not commonly present signs and symptoms that alert to the severity of complications.(1) Thus, its prevention, diagnosis and control is an attribution of Primary Health Care, which may use measures that do not require a high investment, such as case management (CM), in order to keep the disease under control. According to Mendes,(4) CM is a collaborative process between the patient and the professional case manager which aims to agree goals and try to achieve them with the sole purpose of preventing or delaying the occurrence of associated complications, increasing the individual autonomy that allows decision making in the health situation. Studies have shown evidence on the effectiveness of CM developed by nurses for reducing risk factors and decreasing blood pressure levels, as well as improving adherence to the therapeutic regimen.(5-9)

Case management actions also have proved beneficial to increase knowledge about the disease and self-management of treatment.(10) They reduce the number of hospitalizations and aggravations that require emergency care, impacting the improvement of quality of life.(11) The results of the systematic review(12) confirmed that CM is the most frequent intervention in the chronic care model. It has been associated with significant improvements, especially in relation to diabetes and hypertension. Although publications consider the case manager nurse’s role important to control blood pressure levels, no systematic review published in the Cochrane, JBI Database of Systematic Reviews and Implementation Reports or PROSPERO Library, to our knowledge, has examined the effectiveness of nursing CM in the control of hypertension. This gap motivated the development of the present research, whose aim was to verify the effectiveness of nursing CM in primary care compared to usual care, already offered by the health system, in controlling blood pressure in adults with hypertension.

Because it is a chronic disease directly influenced by eating habits, levels of physical activity and adherence to treatment, it was decided to verify whether the literature addressed the influence of CM on these outcomes and blood pressure levels. The review question is: what is the effectiveness of nursing CM versus usual care in primary health care for improving blood pressure in adults over 18 years with hypertension?

### Inclusion criteria

Participants. This review considered studies that included adult patients (over 18 years old) diagnosed with hypertension, with or without other concomitant chronic diseases and followed-up by a case manager nurse as part of a multiprofessional team.

Intervention. This review considered studies that evaluated the effectiveness of case management carried out by nurses and a multidisciplinary team in improving blood pressure. Techniques involving follow-up, monitoring, and health interventions using call centers, tele-nursing, home visits and/or nursing consultations were considered as CM. All studies in which the only systematic difference between the groups was the presence or absence of case management were included; thus, studies with more than two arms were excluded.

Comparators. This review considered studies that compared the intervention to usual care already offered by primary health care centers, which involved the assistance of the health team, with actions that were already part of the normal schedule for patients diagnosed with hypertension, without adding extra activities, only the monitoring regular.

Outcomes. This review considered studies that included the following outcome measures: (i) Blood pressure in mmHg;(1) (ii) Improvement in body mass index (BMI) measured as weight/height^2^ (kg/m^2^);(1) (iii) Increased physical activity measured as time spent performing physical activity each day;(iv) Improvements in lipid profile measured by laboratory examination values of total cholesterol, or high-density lipoprotein, or low-density lipoprotein, or triglycerides; (v) Medication compliance assessed through self-report questionnaires, like the one described by Morisky et al.,(13) the Brief Medication Questionnaire;(14) (vi) Quality of life measured through validated generic or specific questionnaires, such as the hypertension quality of life questionnaire - short form,(15) the Short Form-36 Health Survey;(16) (vii) Smoking cessation assessed through data collection tools, questionnaires or patient self-reports, and eventually assessed by serum cotinine levels; (viii) Financial implications analyzed through cost differences (case management versus usual care).

Types of studies. This review considered study with experimental designs, including randomized and non-randomized controlled clinical trials published in Portuguese, English or Spanish between the years 1990 and 2018. The time limitation is due to the fact that CM has become a strategy used more frequently since the 1990s, due to the rising costs of complex treatments.(17)

## Methods

This systematic review was conducted in accordance with the Joanna Briggs Institute methodology for systematic reviews of effectiveness evidence.(18) This review was conducted in accordance with an a priori protocol(19) (PROSPERO CRD42019112762 - Registration number).

Search strategy. Aiming to find both published and unpublished studies. A three-step search strategy was used in this review. First, an initial limited search of Pubmed an Cinahl was undertaken followed by analysis of the text words contained in titles and abstracts and the index terms used to describe the articles. The search strategy, including all identified keywords and index terms, was adapted for each included information source, and a second search was undertaken on July 12, 2018, to august 30, 2018.

PubMed

#1 (“adult”[MeSH Terms]) AND “hypertension”[MeSH Terms]) OR “hipertenso”[Title/Abstract]) OR “adult”[Title])

#2 ((((((((“case management”[MeSH Terms]) OR “case managers”[Title/Abstract]) OR “managed care programs”[MeSH Terms]) OR “patient care planning”[MeSH Terms]) OR “house calls”[MeSH Terms]) OR “office visits”[MeSH Terms]) OR “visitas a pacientes”[Text Word]))

#3 (((“nurses”[MeSH Terms]) OR “nursing”[MeSH Terms]) OR “nurse/case manager”[Title/Abstract]) OR “nurse/case management”[Title/Abstract]

#4 OR/2-3

#5 ((((((((((((((((((((((((“random allocation”[MeSH Terms] OR “randomized clinical trial”[Abstract]) OR “randomized clinical trial”[Title]) OR “randomized controlled trial”[Abstract]) OR “randomized controlled trial”[Title]) OR “randomized”[Abstract]) OR “randomized”[Title]) OR “randomised”[Abstract]) OR “randomised”[Title]) OR “clinical study”[Abstract]) OR “clinical study”[Title]) OR “clinical trial”[Abstract]) OR “clinical trial”[Title]) OR “clinical trials as topic”[MeSH Terms]) OR “randomly”[Abstract]) OR “trial”[Abstract]) OR “trial”[Title]) OR “groups”[Title]) OR “groups”[Abstract]) OR “placebos”[MeSH Terms]) OR “controlled clinical trial”[Abstract]) OR “controlled clinical trial”[Title] OR “randomised”)))))

# 6 AND/1,4-5 (Filters: Humans) (“1990”[Date - Publication])

Information sources. The databases searched included Pubmed, Cinahl, Lilacs, Web of Science, Scopus, Academic Search Premier, Cochrane Library, WHO Trials. Sources of unpublished studies and gray literature searched included the Directory of Open Acess Journal (DOAJ), CAPES System Portal, Open Gray, European Union Clinical Trial, Proquest Dissertations and Theses, DART Europe E-thesis Portal, World Cat and Electronic Thesis Online System (Ethos). The results obtained in each search in the databases were electronically imported to the Mendeley reference manager (Elsevier), and duplicate records were removed before screening. All the identified studies were evaluated according to the eligibility criteria, based on the information presented in the titles and abstracts. Studies were accessed and assessedin full length for compliance with the eligibility criteria when titles and/or abstracts were not clear/did not have sufficient details to support a decision on their inclusion or exclusion.

Study selection. Following the search, all the identified citations were collated and uploaded into the Mendeley reference manager (Elsevier) and duplicates were removed. Titles and abstracts were screened by two independent reviewers for assessment based on the inclusion criteria for the review. Potentially relevant studies were retrieved in full length and their citation details imported into the Joanna Briggs Institute System for the Unified Management, Assessment and Review of Information (JBI-SUMARI) (Joanna Briggs Institute, Adelaide, Australia). Any disagreements that arose between the reviewers were resolved through discussion, or with a third reviewer.

Assessment of methodological quality. Eligible studies were critically appraised by two independent reviewers at the study level (ATM and JPA) for methodological quality in the review using standardized critical appraisal instrument from the Joanna Briggs Institute.(20) Any disagreements that arose between the reviewers were resolved through discussion, or with a third reviewer (MFM).

Data extraction. Data were extracted from the studies included in the review by two independent reviewers using the standardized Joanna Briggs Institute data extraction tool.(20) The data extracted included specific details about the intervention and time of follow-up, number of participants, comparison group, and results of interest to the review question [systolic (SBP) and diastolic (DBP) blood pressure, BMI, knowledge about hypertension, treatment adherence, abdominal circumference (CA), lipid profile, smoking, consumption of fruits, salt, and alcohol, and physical activity]. Any disagreements that arose between the reviewers were resolved through discussion, or with a third reviewer.

Data synthesis. The results were synthesized using the JBI-SUMARI tool,(20) which were expressed as continuous variables (means and standard deviations), when available, and analyzed. After the analysis, wide heterogeneity was identified for the main outcomes (SBP and DBP) and thus the results of similar outcomes were not grouped in statistical meta-analysis. A narrative and tabular format was used to synthesize and present the results of this review.

Assessment of certainty of evidence in the findings. The Grading of Recommendations, Assessment, Development and Evaluation (GRADE) approach for grading the certainty of evidence followed.(21) The certainty of evidence can be classified high, moderate, low or very low. High certainty: the true effect lies close to that of the estimate of the effect. Moderate certainty: the true effect is likely to be close to the estimate of the effect, but there is a possibility that it is substantially different. Low certainty: the true effect may be substantially different from the estimate of the effect and, Very low certainty: the true effect is likely to be substantially different from the estimate of effect.(21) A Summary of Findings (SoF) was created using GRADEPro GDT (McMaster University, ON, Canada). The SoF presents the following information when appropriate: absolute risks for treatment and control, estimates of relative risk, and a ranking of evidence quality based on the risk of bias, directness, heterogeneity, precision and risk of publication bias of the results of the review. The outcomes reported in the SOF included the PAS and PAD (main outcomes).

## Results

**Figure 1:**
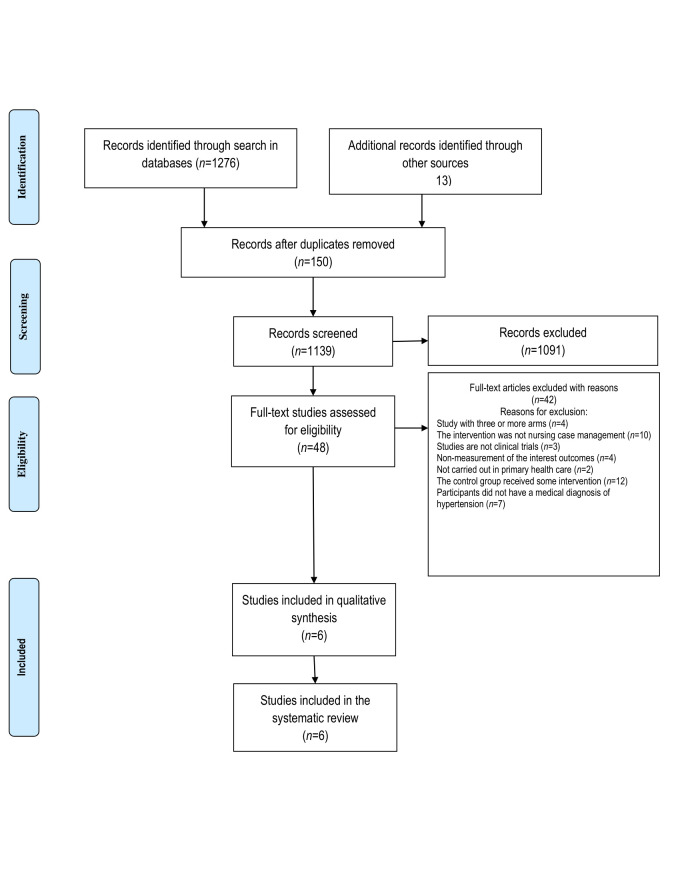
Search results and study selection and inclusion process(22)

Study inclusion. The initial search for commercially published articles and gray literature resulted in a total of 1289 studies. Forty-eight articles remained after removing duplicates and analyzing the titles and abstracts. They were read in full length, and 42 were excluded for not meeting the eligibility criteria. The six remaining articles were critically analyzed using standardized instruments provided by JBI-SUMARI. The main reason for exclusion of articles in this stage - a total of 13 articles - was the fact that none of the study groups received only usual care (Figure 1).

Methodological quality. The reviewers, in teams of two, independently assessed the methodological quality. Despite methodological limitations, none of the six studies undergoing critical appraisal were excluded from the review, because all obtained scores above 60% for the evaluated items. Table 1 outlines the critical appraisal scores for the studies. The assessment of evidence quality and the strength of recommendation of the results obtained through the GRADEPro GDT are presented for the main outcomes of interest in this review (SBP and DBP) according to time of follow-up (6 and 12 months). In none of the studies the participants or researchers who performed the intervention were blind to treatment assignment, or this information was not even described. As for the evaluators, only two studies(23^,^^24^) report that they were blinded, avoiding detection bias. In one of the articles(25) it was not possible to identify how the allocation of participants was made and in another,(7) this item was not respected. This may be related to the nature of the intervention that did not allow the participant or the nurse to be unaware of the division of the groups. The factors that influenced the reduction of evidence quality were mainly the presence of risk of bias, inconsistency related to high heterogeneity, and imprecision due to the reduced sample.

**Table 1:** Critical appraisal results of eligible studies

Studies	Q1	Q2	Q3	Q4	Q5	Q6	Q7	Q8	Q9	Q10	Q11	Q12	Q13	Total
Leiva *et al*. 2014(23)	Y	Y	Y	N	N	Y	Y	Y	Y	Y	Y	Y	Y	11/13
Beune *et al*. 2014(24)	Y	Y	Y	N	N	Y	Y	Y	Y	Y	Y	Y	Y	11/13
Cicolini *et al*. 2014(6)	Y	Y	Y	U	U	U	Y	Y	Y	U	U	Y	Y	8/13
Dean *et al*. 2014(7)	Y	N	Y	N	N	N	Y	Y	Y	Y	Y	Y	Y	9/13
Guirardo *et al*. 2011(25)	U	U	Y	U	U	U	U	Y	Y	Y	Y	Y	Y	7/13
Tonstad *et al*. 2007(26)	Y	Y	Y	U	N	N	Y	N	N	Y	Y	Y	Y	8/13
%	83.3	66.6	100.0	0.0	0.0	33.3	83.3	83.3	83.3	83,3	83.3	100.0	100.0	

Y = Yes, N = No, U = Unclear. JBI critical appraisal checklist for randomized controlled trials: Q1 = Was true randomization used for assignment of participants to treatment groups?; Q2 = Was allocation to treatment groups concealed?; Q3 = Were treatment groups similar at baseline?; Q4 = Were participants blind to treatment assignment?; Q5 = Were those delivering treatment blind to treatment assignment?; Q6 = Were outcome assessors blind to treatment assignment?; Q7 = Were treatment groups treated identically other than the intervention of interest?; Q8 = Was follow-up complete, and if not, were strategies to address incomplete follow-up utilized?; Q9 = Were participants analyzed in the groups to which they were randomized?; Q10 = Were outcomes measured in the same way for treatment groups?; Q11 = Were outcomes measured in a reliable way?; Q12 = Was appropriate statistical analysis used?; Q13 = Was the trial design appropriate, and any deviations from the standard RCT design (individual randomization, parallel groups) accounted for in the conduct and analysis of the trial?

Characteristics of included studies. All studies were carried out in primary care centers on the European continent, two in Spain,(23^,^^25^) one(24) in the Netherlands, one(6) in Italy and one(7) in England. However, one of them(24) was performed on a specific population of Ghanaians and Surinamese. As for the division by sex, all studies included participants of both sexes in both groups (control and intervention). The average percentage of men in the control groups was 49.7% and in the intervention groups 40.8%. As for age, the participants were aged between 18 and 80 years in two studies,(23^,^^25^) between 30 and 69 years in one study,(26) over 18 years in one study,(7) over 20 years in one study(24) and there was no age limit in one study.(6) The mean age per group, but not per sex, could be identified. The mean age of participants was 59.6 years in the intervention group [min 53.3,(24) max 64.5(23) ] and 60 years in the control group [min 54.6,(24) max 66.7(23) ]. Hypertension was defined in all articles as SBP greater than or equal to 140 mmHg and DBP greater than or equal to 90 mmHg. There was heterogeneity in interventions, frequencies of activities developed, and comparisons. Detailed descriptions of interventions can be seen in Table 2 and a summary of the analyzed outcomes and measurement methods in Table 3.

**Table 2:** Characteristics of the studies included in the sample

Study	Synthesis of intervention	Duration and frequency of intervention	Comparison	Outcomes
**Leiva *et al*. 2014(**23)	Divided into 5 components: 1 - A motivational interview based on the Health Belief Model; 2 - Reminders to take the medication; 3 - Family support; 4 - Blood pressure measurements and reminders; 5 - simplification of the therapeutic regime.	Duration: 12 months; Follow-up frequency: 1, 3 and 9 months.	Usual care	SBP and DBP BMI Adherence to treatment/lifestyle Cholesterol (total, LDL, HDL)
**Beune *et al*. 2014**(24)	Usual care plus three counseling sessions using culturally adapted educational materials and, if necessary, referrals to walking clubs and healthy food stores.	Duration: 6 months; Follow-up frequency: 2 weeks, 8 weeks and 20 weeks.	Usual care	SBP and DBP BMI Adherence to treatment/lifestyle
**Cicolini *et al*. 2014**(6)	Usual care, follow-up visits with nurses, and reminders by email and phone calls.	Duration: 6 months; Follow-up frequency: 1, 3 and 6 months. Every day, participants filled out a self-assessment form for treatment adherence and followed an educational program.	Usual care	SBP and DBP BMI Adherence to treatment/lifestyle Cholesterol (total, LDL, HDL) Triglycerides Smoking Alcohol consumption, fruit and vegetable consumption, salt intake, and physical activity
**Dean *et al.* 2014**(7)	Medication review, follow-up with motivational interviews where nurses encouraged changes in habits, and phone calls.	Duration: 6 months; Follow-up frequency: monthly.	Usual care	SBP and DBP
**Guirardo *et al*. 2011**(25)	Four visits adapted according to the needs of the patients. Guidelines were used and leaflets were provided containing information on prescription drugs, dosage and schedule, as well as basic advice on how to maximize treatment schedules.	Duration: 12 months; Follow-up frequency: 1, 3, 6 and 12 months.	Usual care	SBP and DBP BMI Knowledge about hypertension Adherence to treatment/lifestyle
**Tonstad *et al*. 2007**(26)	Monthly meetings with nurses, promotion of changes in lifestyle based on the behavioral self-management model. Individual guidelines were reinforced every month.	Duration: 6 months; Follow-up frequency: monthly.	Usual care with the primary care physician.	SBP and DBP BMI Abdominal circumference Cholesterol (total, LDL, HDL) Triglycerides Glucose

**Table 3:** Summary of analyzed outcomes and measurement methods

Outcome	Study	Evaluation mode
SBP and DBP	Cicolini *et al*. 2014(6);	The device used for measurement is not described.
SBP and DBP	Tonstad *et a*l. 2007(26)	The device used for measurement is not described.
SBP and DBP	Leiva *et al*. 2014(23);	Atomatic device (Omrom-705 IT/Omrom 705 CP)
SBP and DBP	Beune *et al*. 2014(24)	Atomatic device (Omrom-705 IT/Omrom 705 CP)
SBP and DBP	Dean *et al*. 2014(7)	Computerized method
SBP and DBP	Guirardo *et al*. 2011(25)	Mercury sphygmomanometer (average of two measurements with an interval of 2 minutes).
BMI	Beune *et al*. 2014(24);	Weight divided by height in meters
BMI	Guirardo *et al*. 2011(25)	Weight divided by height in meters
BMI	Cicolini *et al*. 2014(6)	Evaluated through a questionnaire validated during a previous cluster randomized trial
BMI	Tonstad *et al*. 2007(26)	Not described
BMI	Leiva *et al*. 2014(23)	Not described
Knowledge about hypertension	Guirardo *et al*. 2011(25)	Batalla test
Adherence to treatment/lifestyle	Guirardo *et al*. 2011(25)	Haynes-Sackett and Morisky-Green questionnaires
Adherence to treatment/lifestyle	Cicolini *et al*. 2014(6);	Morisky-Green questionnaire
Adherence to treatment/lifestyle	Beune *et al.* 2014(24)	Morisky-Green questionnaire
Adherence to treatment/lifestyle	Leiva *et al*. 2014(23)	Retrospective evaluation and determination by the ratio between the number of drugs obtained for the period and the number of days evaluated (6 months)
Abdominal circumferenc	Tonstad *et al*. 2007(26)	Manual
Cholesterol (total, LDL, HDL)	Cicolini *et al*. 2014(6);	Blood sample (automated equipment)
Cholesterol (total, LDL, HDL)	Tonstad *et al*. 2007(26)	Blood sample (automated equipment)
Cholesterol (total, LDL, HDL)	Leiva *et al*. 2014(23)	Form of measurement is not reported, only the parameters used are described.
Triglycerides	Cicolini *et al*. 2014(6);	Blood sample (automated equipment)
Triglycerides	Tonstad *et al*. 2007(26)	Blood sample (automated equipment)
Glucose	Tonstad *et al*. 2007(26)	Automated equipment (Hitachi 911)
Smoking	Cicolini *et al.* 2014(6)	Evaluation through a questionnaire validated during a previous cluster randomized trial.
Alcohol consumption, fruit and vegetable consumption, salt intake, and realization of physical activity	Cicolini *et al*. 2014(6)	Evaluated through a questionnaire validated during a previous cluster randomized trial.

### Review findings

The reading of the selected articles revealed a significant reduction in SBP in two studies,(6^,^^7^) and in DBP in three studies (6^,^^7^^,^^24^) and such reductions were favorable for the intervention group (Table 4). All studies that showed statistical significance were those with a six-month follow-up. Noteworthy is the study by Guirardo et al.(25) which did not present the mean values of SBP and DBP at the end of the study, after a 12-month follow-up, showing only the average difference without a significance test. BMI was another outcome analyzed in two studies, as shown in Table 5.

**Table 4:** Results of studies analyzing SBP and DBP

Study/Groups	Sample	Baseline	End	p-value ^a^	Baseline	End	***p*-value**
**Beune *et al*. 2014**(24)	SBP	SBP	SBP	SBP	DBP	DBP	DBP
Intervention	71	156.7±12.26	146.8±16.23	0.119^a^	91.02±9.61	85.3±10.93	0.009^a^
Usual care	68	155.2±10.69	148.9±13.25	0.119^a^	89.60±9.36	87.9±9.53	0.009^a^
**Cicolini *et al*. 2014**(6)							
Intervention	100	150±11	135±8	< 0.001^b^	87.5±5.7	76.4±5.8	< 0.001^b^
Usual care	98	153±12	143±6	< 0.001^b^	88.6±2.3	81±3.6	< 0.001^b^
**Dean *et al*. 2014**(7)							
Intervention	144	154.2±17.7	142±15.6	0.021^c^	85.6±11.6	79.4±11.1	0.004^c^
Usual care	169	152.9±14.0	146.1±18.9	0.021^c^	85.5±1.7	82.6±11.8	0.004^c^
**Tonstad *et al*. 2007**(26)							
Intervention	29	157±9	147±9	>0.05^c^	94±6	91±8	>0.05^c^
Usual care	16	153±9	143±10		71±10	92±8	>0.05^c^
**Leiva *et al*. 2014**(23)							
Intervention	115	156.3±15.1	151.3±18.3	0.208 ^c^	84.7±10.7	83.4±11.1	0.405 ^c^
Usual care	105	155.5±13.4	153.7±16.8	0.208 ^c^	83.6±10.3	83.6±10.3	0.405 ^c^
**Guirardo *et al*. 2011**(25)							
Intervention	515	140.9±22.8	-	-	82.5±8.9	-	-
Usual care	481	139.3±17.3	-	-	82.2±8.8	-	-

Note: (a) Linear regression analysis; (b) t-test for normally distributed continuous variables; Kruskal-Wallis test for non-normally distributed continuous variables; (c) t-test

**Table 5:** Results of studies analyzing BMI, cholesterol and triglyceride levels

Study/Groups	Sample	Baseline	End	***p*-value**
BMI				
**Cicolini *et al*. 2014(**6)				
Intervention	100	29±6.7	26.5±5.1	< 0.001 ^a^
Usual care	98	26.5±5.1	27.7±4.6	< 0.001 ^a^
**Tonstad *et al*. 2007**(26)				
Intervention	29	27.7±4	27.9±3.9	>0.05 ^b^
Usual care	16	28.6±3.7	29±4	>0.05 ^b^
Cholesterol levels				
**Cicolini *et al*. 2014**(6)	**Total cholesterol *(mg/dL)***	**Total cholesterol *(mg/dL)***	**Total cholesterol *(mg/dL)***	
Intervention	100	265±64	205±40	< 0.001 ^a^
Usual care	98	251±59	218±32	< 0.001 ^a^
**Tonstad *et al*. 2007**(26)	**Total cholesterol *(mmol/l)***	**Total cholesterol *(mmol/l)***	**Total cholesterol *(mmol/l)***	
Intervention	29	6.5	6.3	>0.05 ^b^
Usual care	16	6.2	5.9	>0.05 ^b^
Triglyceride levels				
**Cicolini *et al*. 2014**(6)	**Triglyceride level *(mg/dL)***	**Triglyceride level *(mg/dL)***	**Triglyceride level *(mg/dL)***	
Intervention	100	166±40	142±24	0.12^a^
Usual care	98	177±34	160±37	0.12^a^
**Tonstad *et al*. 2007**(26)	**Triglyceride level *(mmol/l)***	**Triglyceride level *(mmol/l)***	**Triglyceride level *(mmol/l)***	
Intervention	29	1.97±2.16	1.56±1.40	0.03^b^
Usual care	16	1.93±1.39)	2.08±1.30	0.03^b^

Note: (a) t-test for normally distributed continuous variables; Kruskal-Wallis test for non-normally distributed continuous variables; (b) t-test

In addition to the two studies mentioned above, it was found that three others(23-25) measured BMI values, but did not compare the outcome at baseline and end moments through statistical tests. The study by Guirado et al.(25) pointed out the average change between the first and the last measurement, without stating whether the value was significant. Regarding treatment adherence, three studies(6^,^^24^^,^^25^) evaluated the outcome using the Morisky-Green questionnaire. One of them(6) used only one question of the instrument (Did you take your medication yesterday?) and Guirardo et al.(25) also assessed adherence with the Haynes-Sackett questionnaire.

Leiva et al.(23) measured adherence by counting medications and not by a specific instrument like the other studies. However, the research did not compare baseline and end values after the intervention. Guirardo et al.(25) evaluated adherence to treatment through the percentage of people who were adherent to treatment at the initial visit and then presented the percentages of adherence changes for both questionnaires (Haynes-Sackett and Morisky-Green) and identified an increase in treatment adherence assessed by the Morisky-Green Test of 9.6% (95% CI: 5.5-13.6) in the intervention group and 8.8 (95% CI: 4.9-12.6) in the usual care group. The studies by Beune et al.(24) and Cicolini et al.(6) demonstrated an increase in adherence to treatment in both groups. Cicolini et al.(6) analyzed adherence by domains, namely: complete adherence to the drug, dose and hours of therapy. There was an improvement in the percentage of adherence to treatment for all domains in both groups.

Beune et al.(24) presented the results in terms of averages. Higher scores in the questionnaire indicated greater adherence to treatment. Thus, the average score at baseline was 5.99 in the intervention group, reaching 6.49 at the end of the study, whereas, these values were 5.59 and 6.24, respectively, in the usual care group. The total cholesterol was an outcome assessed and compared in two studies.(6^,^^26^) Both showed a reduction in cholesterol levels after a six-month follow-up, with a significant reduction in Cicolini et al.(6) (Table 5).

Cicolini et al. (6) compared LDL-cholesterol levels and identified a significant reduction in the intervention group by the end of the follow-up when compared to the change that occurred in the usual care group (p<0.001). Tonstad et al.(26) measured HDL-cholesterol levels and found a reduction, although not significant, in both groups after follow-up.

Leiva et al.(23) evaluated total cholesterol only for the the purpose of characterization of the sample at baseline. Two clinical trials(6^,^^26^) evaluated triglyceride levels as outcome at the beginning and end of the follow-up; one observed a significant reduction in the intervention group(26) and the other showed a reduction in both groups.(6)

The waist circumference was an outcome assessed by Tonstad et al.(26) showing no improvements at the end of the follow-up in either of the groups. This study also evaluated the glucose outcome. It was observed that glucose levels after six months of intervention decreased in the intervention group and increased in the control group, but these differences were not significant. The smoking outcome was assessed in the clinical trial by Cicolini et al.(6) There were significant improvements only for the intervention group in the first three months of follow-up, and this difference was confirmed at the end of the six months. Leiva et al.(23) evaluated smoking only for baseline sample characterization.

In Cicolini et al.(6) fruit consumption and physical activity were evaluated. There was a significant increase of fruit consumption in the intervention group, with a consequent decrease in the percentage of people who had low fruit consumption per day. With regard to physical activity, at the end of the CM actions, a significant increase in the time of physical activity performed by the intervention group was detected. Alcohol and salt consumption was also evaluated in the clinical trial of Cicolini et al.(6) A significant decrease in the number of alcohol doses per day consumed by the intervention group was observed at the end of the treatment, but no significant differences in salt consumption were seen. The included studies did not analyze the influence of CM on quality of life, nor the financial implications in relation to usual care.

## Discussion

The purpose of this review was to synthesize the best available evidence on the effectiveness of nursing CM in primary health care, compared to usual care, in improving hypertension in hypertensive adults over 18 years of age. The main challenge was related to the type of intervention, which was considered complex because different components were involved. The heterogeneity of the interventions and assessment methods limited the comparisons of results and demonstrated that there is a variety of possible readings about what it means to be a case manager nurse among hypertensive patients, and what strategies can be used for this purpose.

All studies included in this review(6^,^^7^^,^^23^-^26^) applied CM with several activities such as home visits, telephone contact, reminders about the therapeutic regime, motivational interviews, counseling with culturally adapted educational materials, and monthly meetings with nurses. It was found that two studies with duration of six months(6^,^^7^) showed a significant reduction in SBP and DBP over time for the intervention group, one of which(6) applied CM through visits for follow-up by nurses, as well as email and phone reminders. Dean et al.(7) carried out follow-up by nurses with motivational interviews to encourage changes in habits, phone calls and medication reviews.

Although two studies showed significant results of the intervention in the SBP and DBP outcomes, Beune et al (24) reported a greater reduction in blood pressure levels and Tonstad et al.(26) demonstrated a similar reduction in SBP. In both studies, a greater reduction in DBP was obtained for the intervention group. According to the GRADE approach for assessing the certainty of evidence, a low degree of certainty was found. This fact was mainly due to the risk of bias,(6^,^^7^^,^^24^^,^^26^) and serious inaccuracy due to the sample size. (24^,^^26^) Despite the absence of significant results in the comparison between reductions in blood pressure levels in both groups, the 12-month study of Leiva et al.(23) found higher values for the intervention group. However, another study(25) with the same follow-up time found greater reduction in DBP for the intervention group, although SBP was reduced in the control group.

Regarding the quality of the evidence analyzed according to the GRADE approach, it was found that the degree of certainty was low for the SBP outcome due to the risk of bias and inconsistency (heterogeneity above 75%). For the DBP outcome, the degree of certainty was moderate due to the observed risk of bias.

BMI was assessed between the groups in three articles,(6^,^^25^^,^^26^) with only one of them(6) showing a significant reduction in this outcome for the group followed-up with CM, while the other studies showed an increase in values. Regarding adherence to treatment, although different instruments were used to measure this outcome,(6^,^^24^^,^^25^) a fact that hinders comparisons, all showed improvement in treatment adherence among people followed-up with CM. Total cholesterol had a significant reduction in the intervention group in the six-month studies.(6^,^^26^) As for triglyceride levels, only Tonstad et al.(26) reported significant improvement for the group followed-up with CM.

Tonstad et al.(26) was the only study assessing waist circumference and glucose, without significant improvements for the intervention group. Although only one of the articles evaluated these two outcomes, both are risk factors for hypertension and must be a target of intervention for primary care nurses to reduce complications related to the disease.

Lifestyle changes such as smoking cessation, increased fruit consumption, realization of physical activity, and decreased salt consumption were assessed by Cicolini et al.(6) which obtained significant improvements in all outcomes. It is known that lifestyle changes together with the use of the prescribed therapy are key points for the control of hypertension(1).

The strong point of this review is the inclusion of literature from a wide variety of databases and gray literature, as well as the inclusion of studies published in several languages. The limitations of this review are the heterogeneity of the data and the quality of the articles which prevented a meta-analysis. Although comprehensive, it is possible that smaller potentially eligible studies have been published in different languages and were not captured by the search strategies employed here.

Conclusions. Efectiveness of nursing CM for adults with hypertension treated in primary care was not consistently detected in this review. However, some clinical trials reported positive results in terms of reduced blood pressure, improved treatment adherence, and improved cholesterol levels. The wide variety of actions used in CM, as well as the different ways of measuring the analyzed outcomes, prevented a statistical comparison of studies.

Recommendations for practice. The findings of this review demonstrated with low and very low certainty that case management performed by six months had a positive impact in reducing DBP and SBP respectively. Although there is no high evidence, it is considered that CMs performed in primary health care can assist in the planning of health actions, health promotion activities, disease prevention, reduction of comorbidities, and control of chronic diseases, as for example control of hypertension.

Recommendations for research. Many studies address the importance of monitoring by nurses to reduce pressoric levels, but the methodologies used in the studies do not provide strong evidence to confirm this statement. More randomized clinical trials with methodological quality and expressive samples need to be performed to confirm or refute the effect of case management for people with hypertension in primary care.
